# Using Wearable Cameras to Categorize the Type and Context of Screen-Based Behaviors Among Adolescents: Observational Study

**DOI:** 10.2196/28208

**Published:** 2022-03-21

**Authors:** George Thomas, Jason A Bennie, Katrien De Cocker, Fitria Dwi Andriyani, Bridget Booker, Stuart J H Biddle

**Affiliations:** 1 School of Allied Health Curtin University Bentley Australia; 2 Centre for Health Research University of Southern Queensland Springfield Central Australia; 3 Department of Movement and Sports Sciences University of Gent Gent Belgium; 4 Department of Sports Education Yogyakarta State University Yogyakarta Indonesia; 5 Institute for Positive Psychology and Education Australian Catholic University North Sydney Australia

**Keywords:** adolescent, screen time, smartphone, television

## Abstract

**Background:**

Automated wearable cameras present a new opportunity to accurately assess human behavior. However, this technology is seldom used in the study of adolescent’s screen exposure, and the field is reliant on poor-quality self-report data.

**Objective:**

This study aimed to examine adolescents’ screen exposure by categorizing the type and context of behaviors using automated wearable cameras.

**Methods:**

Adolescents (mean age 15.4 years, SD 1.6 years; n=10) wore a camera for 3 school evenings and 1 weekend day. The camera captured an image every 10 seconds. Fieldwork was completed between February and March 2020, and data were analyzed in August 2020. Images were date and time stamped, and coded for screen type, content, and context.

**Results:**

Data representing 71,396 images were analyzed. Overall, 74.0% (52,842/71,396) of images contained screens and 16.8% (11,976/71,396) of images contained multiple screens. Most screen exposures involved television sets (25,950/71,396, 36.3%), smartphones (20,851/71,396, 29.2%), and laptop computers (15,309/71,396, 21.4%). The context of screen use differed by device type, although most screen exposures occurred at home (62,455/64,856, 96.3%) and with solitary engagement (54,430/64,856, 83.9%). The immediate after-school period saw high laptop computer use (4785/15,950, 30.0%), while smartphone use (2059/5320, 38.7%) peaked during prebedtime hours. Weekend screen exposure was high, with smartphone use (1070/1927, 55.5%) peaking in the early morning period and fluctuating throughout the day.

**Conclusions:**

There was evidence for high screen use during the after-school and weekend period, mostly through solitary engagement, and within the home environment. The findings may inform the basis of larger studies aimed at examining screen exposure in free-living conditions.

## Introduction

Electronic screens, such as those of smartphones, tablets, and televisions, are ubiquitous in modern society [1]. Systematic reviews and meta-analyses have shown that higher levels of adolescent screen use are associated with detrimental health outcomes, such as increased adiposity [2,3] and depression [4,5], as well as low academic achievement [6]. Others argue that the health effect of screen use is complex [7], and for well-being, it may be negligible [8] or, in some cases, beneficial [9]. To better understand the impact on adolescent outcomes, it is important to use robust methods of measuring screen use [10]. However, the current evidence is limited by several methodological factors.

First, the vast majority of screen use evidence has relied on self-reported data [11]. There is widespread consensus that such reporting of sedentary behavior lacks measurement precision due to recall difficulties and is prone to numerous biases (eg, social desirability) [12]. In addition, traditional self-reported measures of screen use, such as questionnaires and time use diaries, focus primarily on televisions, computers, and video games, and have largely ignored smartphones and tablets, which make up an increasingly large proportion of adolescent discretionary screen use [13,14]. Furthermore, there have been recent increases in newer digital media use among adolescents, such as social networking and online communication [15], which might be replacing television viewing [16]. Therefore, measurement needs to adapt to the modern reality of screen use and be flexible to allow for the incorporation of new technologies as they emerge [1].

Second, there is the issue of *multiscreening*, the simultaneous use of multiple screens, which may have implications for the measurement of screen use. At present, most questionnaires assess each screen use behavior independently and then sum these individual behaviors to calculate *total* screen time. Therefore, this may preclude accurate estimates of an individual’s overall screen exposure if they are using multiple screens concurrently [17]. Given that self-reported and other-reported data indicate that adolescents may be more likely to use multiple screens than any other age group [18,19], it is important to gather information about the patterns of use in this population. This includes examining the task combinations that underpin these patterns, in addition to which media types are typically used for the primary activity or the secondary activity.

Third, most studies have used aggregated *total* screen use measures or have grouped them into broad categories (eg, television and computer). Such methodology fails to investigate the different types of content that may moderate the effects of screen exposure on children’s health, social, and developmental outcomes [20]. When these aspects have been measured, the context of these behaviors is often overlooked, specifically, “when,” “where,” and “with whom” adolescents are using screens. Using aggregates of behavior masks the context specificity of each behavior and thus precludes accurate conclusions about specific behaviors occurring at specific time points and in specific contexts [21]. Such contextual information might be used when designing interventions to inform new policies specifically designed to influence adolescents’ screen use.

Fourth, despite the importance of temporal patterning to better understand the physical activity levels of young people [22], studies that have investigated this aspect of screen use are limited. When temporal patterning is measured, the evidence tends to rely on self-report, such as time use diaries [21,23]. While these allow for the recording of behaviors as well as locations throughout the day [24], the recording of activities relies on the judgement and memory of participants, depending on the time completed. Moreover, time use diaries can also be burdensome for participants, possibly causing involuntary changes in activity behavior throughout the day [23]. It is necessary to identify and corroborate the trends in the temporal, social, and environmental contexts of adolescents’ screen-based behaviors using less obtrusive low-burden device-based measures.

Automated wearable cameras present an emerging opportunity to more accurately assess adolescents’ exposures to screens, including the social and environmental contexts in which they occur [25]. Such cameras have the advantage of monitoring behaviors through a first-person perspective in free-living conditions [26,27]. Human behavior research has increasingly employed this technology, as the devices become smaller, more affordable, and capable of capturing more data [28-32]. For example, wearable cameras have been used to investigate children’s physical activity [33], diet [34], exposure to blue space [35], food and alcohol marketing [36,37], green space, transport, and smoking [38]. Moreover, wearable cameras have been applied to examine adult’s sedentary behaviors [32]. However, few studies on adolescent’s sedentary behaviors have used this technology, with the field being mainly reliant on poor quality self-reported data.

Smith et al recently demonstrated wearable cameras to be a feasible and acceptable method of measuring evening screen exposure among New Zealand adolescents [39]. This study collected 41,734 images across 39 evenings, showing that almost half of the images contained screens, most commonly those of smartphones, while 5% contained multiple screens. However, data were derived largely from nonschool days, owing to examination during study breaks or holidays, and thus, the findings may not reflect adolescents’ typical screen use. For instance, available evidence suggests that adolescents spend over 70% of their after-school time sitting [40], with a large proportion spent using screens [41,42].

To address current gaps in evidence, this study aimed to use automated wearable cameras to examine adolescents’ screen exposure during the evenings and weekends of a typical school term. In particular, we aimed to describe (1) the frequency and the types of devices being used; (2) the content being viewed; (3) the social and environmental context in which such behaviors occur; and (4) the temporal patterning of screen-based behaviors. We also aimed to describe differences in screen time between weekdays and weekend days.

## Methods

### Ethics Approval

Ethical approval was obtained from the University of Southern Queensland Human Research Ethics Committee (H19REA248). In line with international guidelines [43], this study adhered to strict procedures for using wearable cameras in human research. Data were collected between February and March 2020 (before any effects of COVID-19 restrictions) and analyzed in August 2020. Written informed parental and adolescent consents were obtained before data collection.

### Sampling and Recruitment

Ten participants (aged 13-17 years) were recruited from a secondary school in Queensland, Australia. All students in grades 8 to 11 (age 13-17 years; N=100) attended a face-to-face information session in which they were invited to take part in a research study. At the end of the session, the principal investigator (GT) answered questions and provided research packs to adolescents interested in participating (n=17). Written parental and adolescent consents were obtained for 10 adolescents (response rate 59%).

### Measures

#### Sociodemographic Questionnaire

Before data collection commenced, parents were asked to complete a brief questionnaire concerning demographic characteristics, including the highest education level, household income, and employment status.

#### Automated Wearable Cameras

Participants were asked to wear an automated camera (Brinno TLC120) on 4 randomly allocated days (using an online random number generator), including 3 school weekday evenings (all waking hours after returning home from school) and 1 weekend day (all waking hours). The automated camera was programmed to take a picture every 10 seconds. The camera had a weight of 101 g, had a size of 60×60×35 mm, captured a 112° field of view, and did not record audio or video. The battery had a capacity of 6 days when using the 10-second interval. Images were date- and time-stamped. Participants were instructed to wear the camera on an adjustable chest-mounted harness. An information session, facilitated by the first author, provided instructions on how to turn the camera on/off, how to wear the harness, and how to charge the camera, if necessary. Information sheets were also provided to participants that offered examples of when participants should remove or turn the camera off (eg, going to the bathroom or getting undressed). Lastly, a statement of research was handed to participants to help explain the study to third parties (eg, public, friends, and family), if required.

The camera automatically processed images into time-lapse videos (.avi), which were then manually converted into single images (.jpg) using the open-source software FFmpeg (version 4.3). Participants and parents were offered an opportunity to review and delete images before the first author viewed them. To protect participant privacy, the remaining images were securely stored using a password-protected storage server only accessible to the image coders.

### Image Coding

**Figure 1:**
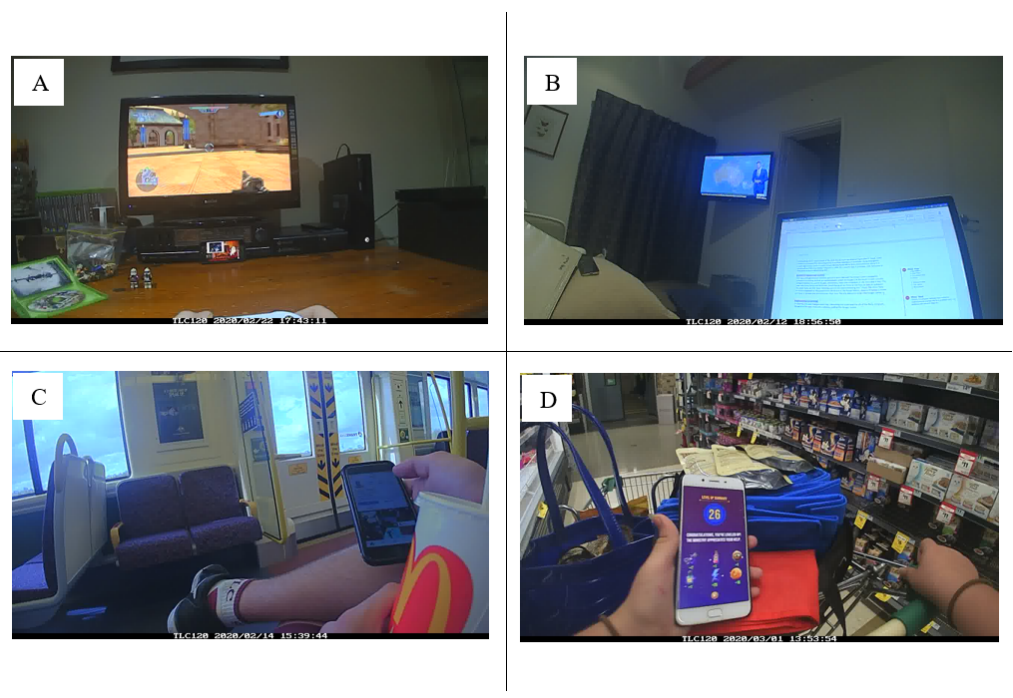
Sample of images and coding of screen-based behaviors. (A) Primary screen: television; content: game > action; content classification: recreational; secondary screen: smartphone; content: TV programs > action animation; content classification: recreational; location: home > living room. (B) Primary screen: laptop computer; content: creative > productivity software; content classification: educational; background screen: television; content: TV programs > action; content classification: recreational; location: home > bedroom. (C) Primary screen: smartphone; content: social media > Instagram; content classification: social; location: transport > public transport; other behavior: food > beverage. (D) Primary screen: smartphone; content: game > simulation; content classification: recreational; location: public > food retail.

Examples of images and coding are presented in Figure 1. Image coding was completed between April and July 2020. Images for each participant were manually coded by the first author in a spreadsheet, based on a pre-established codebook for wearable camera research on children’s screen use. The coding protocol was structured into different annotation groups (Multimedia Appendix 1). For each annotation group, the coder identified all the images and categorized only the screen components specific to that group. Images with multiple screens were coded for multiscreening, detailing the primary, secondary, or background activity. Images with inactive or absent screens were also coded, in addition to blurry or blocked images because of the position of the camera. Obscured images (such as those where the camera was facing the ceiling) in the middle of image sequences containing screens were coded based on the nonobscured preceding and subsequent images. A subset of images (10%) was repeat coded by a second researcher, and interrater reliability was tested using the Krippendorf (*α*) statistic to determine consistency between coders for all coding categories. Interrater reliability was interpreted using the guidelines of Krippendorf [44].

### Data Analyses

IBM SPSS Statistics version 21.0 was used for descriptive analyses. Daily camera wear time was calculated as the total number of minutes the camera was turned on. Captured time (minutes) was the number of images divided by 6 (assuming each image represented 10 seconds). The frequency and percentage of images were calculated for each screen-based device for each annotation group (eg, location and social interaction). Descriptive data are provided to describe the frequency and types of devices being used, the content being viewed, and the social and environmental context in which such behaviors occur. To analyze the temporal patterning and to compare screen-based behaviors between the different evening segments, equal time segments of 3 hours were utilized. For each time period, the frequency of each screen-based behavior was computed, and the percentage of behaviors occurring at that time period has been reported. Temporal data were analyzed and reported separately for weekdays and weekend days because they have different structures and are likely to lead to different behavioral choices. Differences between weekday and weekend screen use were analyzed using the chi-squared test, and expressed as a percentage of images (standardized by weekday and weekend wear times). Based on previously established definitions [45], weekday after-school time segments were defined as follows: “after school to 18:00,” “18:00 to 21:00,” and “21:00 to sleep” (eg, when the camera was removed prior to bedtime).

## Results

### Interrater Reliability

The average reliability between the 2 coders across all categories was acceptable (*α*=.81) [44]. With regard to agreement, the *α* values were .89 for device attention, .86 for device type, .77 for content type, .88 for content classification, .60 for physical setting, .85 for social setting, .84 for social interaction, .72 for co-existing behaviors, and .88 for the uncodable category.

### Sample Characteristics

The characteristics of the participants are presented in Table 1. Five girls and five boys participated, with an average age of 15.4 years (SD 1.6 years). The main language spoken at home was English (9/10, 90%), with an average of 4 persons living in the household. Participants’ parents who responded were mainly mothers (8/10, 80%), married (9/10, 90%), and earning a total annual household income >AUD 78,000 (9/10, 90%; 1 AUD = 0.73 USD), and had completed a university or tertiary qualification (8/10, 80%).

**Table 1:** Characteristics of the sample.

Variable		Value (N=10)
Gender (% female)		50
Parent gender (% female)		80
Age (years), mean (SD)		15.4 (1.6)
Number of people in the household, mean (SD)		4.0 (1.1)
**Main language, n (%)**		
	English	9 (90)
	Other	1 (10)
**Total annual household income (AUD^a^), n (%)**		
	>78,000	9 (90)
	31,200-41,599	1 (10)
**Parents’ highest level of education, n (%)**		
	University or tertiary qualification	8 (80)
	High school	1 (10)
	Year 12 or equivalent	1 (10)
**Parental marital status, n (%)**		
	Married	9 (90)
	Separated/divorced	1 (10)

^a^1 AUD = 0.73 USD.

### Overview of Images

A total of 71,396 images, derived from 30 school weekday evenings and 10 weekend days, were coded and included in the analysis. This represented just under 200 hours of total camera wear time. Multimedia Appendix 2 shows the mean and median (IQR) numbers of images collected, camera wear time, captured time, and screen time per day for weekdays and weekend days. The camera wear time averaged 230.5 minutes on a weekday evening and 508.1 minutes on a weekend day. The camera captured, on average, 1365 images per weekday evening and 3045 images per weekend day, equating to 227.5 minutes and 504.2 minutes of captured time, respectively. Of this, 167.7 minutes were spent, on average, using screens on a weekday evening, and 371.3 minutes were spent on a weekend day. The results showed that there was no significant difference between weekday and weekend screen use (72.7% vs 73.1%, *P*=.23).

### Device and Content Type

Table 2 shows the frequency and percentage of different screens and activities in the entire image set (N=71,396). In total, 52,842 (74.0%) images contained screens. The most common screens were televisions (25,950/71,396, 36.3%), smartphones (20,851/71,396, 29.2%), and laptop computers (15,309/71,396, 21.4%), while fewer images contained tablets (2720/71,396, 3.8%), desktop computers (20/71,396, <1%), and wearable smartwatches (1/71,396, <1%).

The most common activities, as determined by the proportion of images recorded by the wearable camera, differed according to the screen domain. For instance, our data showed that conventional television sets were popular among adolescents, although this comprised mostly playing *action* games (ie, including fighting, shooter, or platform games) via gaming consoles (14,032/25,950, 54.1%), rather than watching traditional *action* television programs (ie, programs with real people or animals), which accounted for less than half of all television occurrences (11,803/25,950, 45.5%). Given this information, the results for “television set” occurrences were described by (1) television set: *television viewing* and (2) television set: *action gaming*. For smartphones, watching television programs through online streaming sites, such as Netflix and YouTube (10,432/20,851, 50.0%), social networking (5642/20,851, 27.1%), and communicating (1618/20,851, 7.8%) constituted the main content types, compared to creative content, such as *productivity software* (eg, Word, Excel, and PowerPoint), which made up 39.5% (6051/15,309) of all laptop computer occurrences. Watching television programs (4763/15,309, 31.1%) and internet use (3409/15,309, 22.3%) also made up a large proportion of content engaged on the laptop computer, while the same content accounted for 51.0% (1387/2720) and 30.3% (825/2720) across all tablet occurrences, respectively. A wearable smartwatch was captured in 1 image, showing the home screen interface. For all desktop computer images (n=20), specific content could not be determined owing to inadequate resolution.

**Table 2:** Description of devices and types of content.

Device^a^, broad content^b^, and specific content^b^				Value, n (%)	
Any screen				52,842 (74.0)	
No screen				18,554 (26.0)	
Multiple screens				11,976 (16.8)	
**Television**				25,950 (36.3)	
	**Television set: gaming (via console)**			14,032 (54.1)	
		Action	14,032 (54.1)		
	**Television set: television viewing**			11,803 (45.5)	
		Action	11,250 (43.4)		
		Action animation	464 (1.8)		
		Animation cartoon	89 (0.3)		
	Unclassifiable			115 (0.4)	
**Smartphone**				20,851 (29.2)	
	**Television programs**			10,432 (50.0)	
		Action	6658 (31.9)		
		Animation cartoon	2794 (13.4)		
		Action animation	980 (9.4)		
	**Social media**			5642 (27.0)	
		Instagram	3153 (15.1)		
		TikTok	2046 (9.8)		
		Snapchat	286 (1.4)		
		Facebook	157 (0.8)		
	**Communication**			1618 (7.8)	
		Instant/text messaging	1182 (5.7)		
		Video chatting	230 (1.1)		
		Calling	206 (1.0)		
	**Creative**			675 (3.2)	
		Camera apps	475 (2.3)		
		Art apps	119 (0.6)		
		Productivity software	81 (0.3)		
	**General**			618 (3.0)	
		Home page, lock screen notifications	618 (3.0)		
	**Internet**			585 (2.8)	
		Browsing	320 (1.5)		
		Article/book/blog	265 (1.3)		
	**Gaming**			555 (2.7)	
		Simulation	405 (1.9)		
		Action	150 (0.7)		
	**Interactive screen media**			436 (2.1)	
		Other	411 (2.0)		
		Unclassifiable	25 (0.1)		
	Unclassifiable			290 (1.4)	
**Laptop computer**				15,309 (21.4)	
	**Creative**			6579 (43.0)	
		Information processing apps	6051 (39.5)		
		Art apps	528 (3.4)		
	**Television programs**			4763 (31.1)	
		Action	4119 (26.9)		
		Animation cartoon	644 (4.2)		
	**Internet**			3409 (22.3)	
		Article/book/blog	3070 (20.1)		
		Browsing	339 (2.2)		
	**General**			409 (2.7)	
		Home page, lock screen notifications	409 (2.7)		
	**Social media**			72 (0.4)	
		TikTok	72 (0.4)		
	**Interactive screen media**			44 (0.3)	
		Other	44 (0.3)		
	Unclassifiable			33 (0.2)	
**Tablet**				2720 (3.8)	
	**Television programs**			1387 (51.0)	
		Action	1387 (51.0)		
	**Internet**			825 (30.3)	
		Article/book/blog	728 (26.8)		
		Browsing	97 (3.6)		
	**Creative**			420 (15.4)	
		Productivity software	252 (9.3)		
		Art apps	168 (6.2)		
	**Communication**			66 (2.5)	
		Instant/text message	66 (2.5)		
	**General**			13 (0.5)	
		Home page, lock screen notifications	13 (0.5)		
	**Interactive screen media**			9 (0.3)	
		Other	9 (0.3)		
**Desktop computer**				20 (0.0)	
	**Unclassifiable**			20 (100.0)	
		Unclassifiable	20 (100.0)		
**Wearable smartwatch**				1 (0.0)	
	**General**			1 (100.0)	
		Home page, lock screen notifications	1 (100.0)		
**Unclassifiable**				5 (0.0)	
	Unclassifiable			5 (100.0)	

^a^For the device variables, the number and percentage of images are based on the total image set (71,936 images).

^b^For the broad and specific content variables, the number and percentage of images are based on the respective device image set (eg, television and smartphone).

### Content Classification

As shown in Table 3, *recreational* activities made up the majority (45,218/64,856, 69.7%) of all screen occurrences, compared with other content classifications, such as *educational* (10,603/64,856, 16.3%) and *social* (7450/64,856, 11.5%) activities. Concerning individual device types, all traditional television viewing was classified as *recreational*, over half of laptop computer activities were *educational* (9361/15,309, 61.1%), and more than a third of smartphone exposure was deemed *social* (7265/20,851, 34.9%). Tablets were commonly used for *recreational* (1627/2720, 59.8%) and *educational* (1014/2720, 37.3%) purposes, although tablets comprised only 3.8% (2720/71,936) of total screen exposure.

**Table 3:** Content classification of adolescents’ screen-based activities.

Device and nature of content			Value, n (%)
**All screens^a^ (n=64,856)**			
	Recreational	45,218 (69.7)	
	Educational	10,603 (16.3)	
	Social	7450 (11.5)	
	Other	1022 (1.6)	
	Unclassifiable	563 (0.9)	
**Television set (n=25,950)**			
	Recreational	25,788 (99.4)	
	Unclassifiable	115 (0.4)	
	Social	47 (0.2)	
**Television set: action gaming (n=14,032)**			
	Recreational	13,985 (99.7)	
	Social	47 (0.3)	
**Television set: television viewing^b^ (n=11,803)**			
	Recreational	11,803 (100.0)	
**Television set: unclassifiable (n=115)**			
	Unclassifiable	115 (100.0)	
**Smartphone (n=20,851)**			
	Recreational	12,369 (59.3)	
	Social	7265 (34.9)	
	Other	615 (2.9)	
	Unclassifiable	374 (1.8)	
	Educational	228 (1.1)	
**Laptop computer (n=15,309)**			
	Educational	9361 (61.1)	
	Recreational	5434 (35.5)	
	Other	393 (2.6)	
	Social	66 (0.4)	
	Unclassifiable	49 (0.4)	
**Tablet (n=2720)**			
	Recreational	1627 (59.8)	
	Educational	1014 (37.3)	
	Social	66 (2.4)	
	Other	13 (0.5)	
**Desktop computer (n=20)**			
	Unclassifiable	20 (100.0)	
**Wearable smartwatch (n=1)**			
	Other	1 (100.0)	
**Unclassifiable (n=5)**			
	Unclassifiable	5 (100.0)	

^a^Based on all screen-based coding interactions (including images with multiple screens).

^b^Comprises action, action animation, and animation cartoon programs.

### Multiscreening

As shown in Multimedia Appendix 3, more than 16% (11,976/71,936, 16.7%) of images contained multiple screens, with the most prevalent combinations of screens being (1) television-smartphone (7324/11,976, 61.2%), (2) smartphone-laptop (2558/11,976, 21.4%), and laptop-television (985/11,976, 8.2%). The majority of multiscreening involved (1) televisions as the primary screen and smartphones as the background screen (5029/11,976, 42.0%) used for *gaming* and watching *television programs*, respectively; (2) smartphones as the primary screen and televisions as the background screen (2285/11,976, 19.1%) used for watching *television programs* and *gaming*, respectively; and (3) smartphones as the primary screen and laptops as the background screen (1465/11,976, 12.2%) used for *social networking* and *internet use*, respectively.

### Physical Setting

As shown in Multimedia Appendix 4, nearly all screen exposures occurred in the home setting (62,455/64,856, 96.3%), such as the *living room* (37,364/64,856, 57.6%) and *bedroom* (19,473/64,856, 30.0%). Concerning individual screen domains, all action gaming (via television set) and the majority of traditional television viewing (10,793/11,803, 91.4%) occurred in the *living room*, whereas laptop computers were commonly used in the *bedroom* (8974/15,309, 58.6%). Smartphones were used in several areas, including the *living room* (8719/20,851, 41.8%) and *bedroom* (7932/20,851, 38.0%), and when in *private transport* (1564/20,851, 7.5%), while the *bedroom* (1709/2720, 62.8%) and *kitchen/dining room* (848/2720, 31.2%) served as popular locations for tablet use.

### Social Setting and Interaction

The social contexts surrounding adolescents’ screen exposure are presented in Multimedia Appendix 5. Most involved no in-person social interaction (54,430/64,856, 83.9%), although this differed by screen domain. In-person social interaction was greater when watching television programs, including *co-viewing* with an adult (1719/11,803, 14.6%) or child (1368/11,803, 11.6%), whereas nearly all action games were played alone (12,915/14,032, 92.0%). For smartphones, 12.8% (2668/20,851) of images involved an adult in the *background*, while fewer in-person social interactions were experienced with laptop computers, tablets, and desktop computers (with 92%-100% of all occurrences engaged in alone).

### Co-existing Behaviors

As shown in Multimedia Appendix 6, the majority of screen use was in isolation (56,656/64,856, 87.4%). Some co-existing behaviors that occurred alongside screen-based behaviors included writing using a *pen and paper* (3873/64,856, 6.0%), eating a *snack* (1755/64,856, 2.7%), or eating a *meal* (1454/64,856, 2.2%), but this varied according to screen domain. Laptop (2430/15,309, 15.9%) and tablet (800/2720, 29.4%) computers were commonly used when writing with a *pen and paper*, while over 10% (1288/11803, 10.9%) of television viewing occurrences involved consuming food (eg, *snack* or *meal*).

### Temporal Patterns

**Figure 2:**
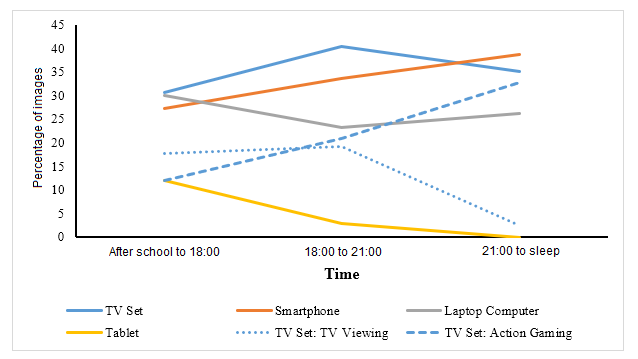
Occurrences of screen-based behaviors during school weekday evenings. TV: television.

For each time period, the frequency of each screen-based behavior was computed. These data represent the percentage of behaviors occurring at that time period; thus, the results reported are necessarily descriptive. Figures 2 (weekdays) and 3 (weekends) compare the temporal patterns of screen-based behaviors.

**Figure 3:**
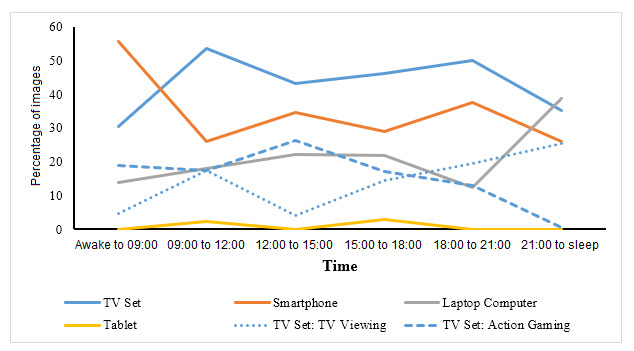
Occurrences of screen-based behaviors during weekends. TV: television.

#### Weekday

Television occurrences were most common in the middle evening segment (18:00-21:00; 6593/16,359, 40.3%). This comprised mostly of playing action games (3440/16,359, 21.0%), which continued to increase into the prebedtime period (≥21:00; 1738/5314, 32.7%), compared to television viewing, which was highest during the middle evening (2875/14,896, 19.3%). During the immediate after-school period (≤18:00), occurrences of laptop computers (4785/15,950, 30.0%) peaked and then consistently decreased throughout the evening. The use of smartphones increased from the early evening period (4337/15,944, 27.2%) through to the prebedtime period (2059/5320, 38.7%). Tablet computers were most common after school (1918/15,950, 12.0%), while no occurrences were captured after 21:00.

#### Weekend

Temporal patterns on the weekend were more varied than during the school weekday evening. Smartphone use peaked (1070/1927, 55.5%) during the time period from waking to 09:00, followed by fluctuating occurrences throughout the day. Television occurrences were fairly consistent across the day, but differed by content domain, with larger occurrences of watching action programs from 18:00 (1671/8569, 19.5%) through to sleep (612/2409, 25.4%%). In contrast, playing action games was common during the afternoon hours, peaking between 12:00 and 15:00 (2016/7694, 26.2%), before declining throughout the evening. Concerning laptop computer use, 38.7% (691/1785) of occurrences were captured during the prebedtime period, doubling that during the previous time period between 18:00 and 21:00 hours. Tablet computers were uncommon during the day, comprising only 2.4% (158/6583) of occurrences in the morning and 2.9% (163/5620) between 15:00 and 18:00.

## Discussion

### Summary and Interpretation of Results

This study aimed to use automated wearable cameras to describe adolescents’ screen exposure. Consistent with a recent systematic scoping review [46], our data indicated that adolescents are exposed to high amounts of screen use. In particular, our data showed a greater percentage of time on smartphones, with engagement in various activities, such as watching programs, social networking, and communicating online, compared to time using television sets and engaging in conventional television viewing. This finding supports the perception that use of newer digital media is increasing, with some displacement of traditional forms of media for adolescents [16]. Indeed, reports show a decline in watching programs on conventional television sets, despite an increase in consuming television content on the internet [47]. This is likely caused by the multiple functions that smartphones offer, including the social and recreational tasks performed online [48]. Moreover, the portability of smartphones allows adolescents to use these devices ubiquitously [49]; almost anywhere in free-living conditions as reported here. Future studies need to determine effective strategies for the responsible use of contemporary screen engagement, paying attention to the use of smartphones [13]. Moreover, these findings have implications for the assessment of television viewing, a common category for screen time measurement. If television programs are watched on television sets, as well as other devices, we may be estimating behaviors incorrectly, depending on the nature of the question asked. Hence, it is possible that there may be conflating of the assessment of behaviors and devices. Better measurement that captures the types of devices used for watching television programs, in addition to the social environmental contexts of such viewing, is warranted.

This study also showed that multiscreening is evident in screen use among adolescents, supporting conclusions from a previous study [50]. Here, we revealed that multiple screens were identified in approximately 17% of images across the entire image set. This rate was more than 3 times the rate reported by Smith et al among adolescents in New Zealand [39]. An important part of understanding multiscreening is examining the combinations of tasks undertaken. Contrary to previous findings based on self-report data [19,51], our data showed that gaming via television together with watching programs on a smartphone was the most common combination of screen exposure. Previously, it has been argued that gaming is harder to combine with another screen because it demands many cognitive capacities and behavioral responses [52]. One explanation for our findings is that although gaming was used concurrently with smartphones, the latter was predominantly used in the background and therefore was less likely to interfere with adolescents’ cognitive demands of gaming. Other possible explanations are that smartphones were used to temper impatience or boredom whilst waiting for a game to load [17,48], or offered an opportunity to socialize with friends whilst watching television shows [48]. Further investigations on why adolescents engage in certain multiscreening behaviors (eg, social functions) are needed to help researchers deliver effective interventions to change screen-based behaviors, if deemed necessary. Models and theories, specific to multiscreening behaviors among adolescents, might also warrant further enquiry.

The home environment may serve as an important setting for interventions that aim to influence adolescents’ screen exposure. Participants in this study spent the majority of their time at home and, as such, engaged in most of their screen time at home. Consistent with previous findings [21], the living room was strongly linked to television viewing. While expected, this finding suggests that if reductions in television viewing are sought, this location may be a target for interventions, such as through environmental restructuring involving reconfiguring seating arrangements or family rules for behaviors while in the room. In addition to the living room, the bedroom may also be an important context for screen use, particularly involving smartphones, tablets, and laptop computers, as shown in the current study. It is possible that adolescents feel they have greater privacy and have fewer interruptions from family members in this setting. This might encourage prolonged recreational use of screens in this location, often whilst sitting or lying down [48]. Indeed, reducing access to screen-based devices in the bedroom has been identified as a facilitator to reduce screen use [53], although this will not guarantee a reduction in sedentary behavior as other sedentary pursuits may be adopted as a substitute.

Corroborating recent qualitative data [41], the present study found that adolescents had very little *in-person* social interactions with others whilst using screens. Such findings support the hypothesis of time displacement for social interaction, that is, more adolescents spending time on digital media and less time on face-to-face social interaction [54]. This has led to concern over the detrimental impacts of screen use on adolescents’ psychological well-being, with some finding links to depression, loneliness, and lower social connectedness [55-57], although the associations might be small and complex [8,56,58]. Others argue that digital media may instead compliment *in-person* social interaction, particularly media involving opportunities to interact online [59]. For instance, despite a lack of *in-person* social interactions observed in this study, it is highly likely that adolescents engaged in numerous online interactions whilst using screens, for example, through online communication or playing interactive video games. The mechanisms of the putative effects of screen use on psychological well-being should be explored further to better understand the impact of lower *in-person* social interactions that might be characteristic of traditional solitary screen use, in addition to higher digital social interactions that might be typical of newer digital media. Such research may need to account for the types of devices as well as physical and social settings.

By investigating the temporal patterns of screen use, we were able to understand the typical schedule of adolescents and identify timeframes when specific screen-based behaviors compete against each other. Similar to previous self-report data [21,23], screen-based behaviors were shown to be prominent throughout the evening, starting immediately after school until the end of the day. As shown in a systematic review [42], the after-school period is linked to high screen use in adolescents. This was particularly apparent for laptop computers, which were likely used to complete educational tasks, such as homework. Despite the sedentary nature of this behavior, such tasks are generally considered to be important and valuable; thus, whether it should be reduced or replaced with other behaviors, such as physical activity, is debatable [23]. This supports the argument that sedentary behaviors should not be viewed in isolation [21].

As for television viewing, this behavior most likely occurred in the middle evening segment, corroborating previous findings using self-report [21]. However, the same behavior reduced rapidly in the prebedtime hours, comprising just 2.4% of all screen occurrences. This may be due to the changes in patterns of media consumption among young people. Here, we found that smartphones peaked in the hours before bedtime, supporting evidence that portable devices are increasingly part of the adolescent sleeping environment [60]. For instance, using data from a large population-based survey of adolescents in Norway (n=9846), Hysing et al showed that approximately 80% of boys and 90% of girls used a cell phone in the hour before going to sleep [61]. Together, these findings may cause a rise in public health concern, especially given the evidence for associations between prebedtime screen use and a number of poor sleep markers (eg, inadequate sleep quantity, poor sleep quality, and excessive daytime sleepiness) [62]. As such, smartphones may be an important target for interventions that aim to mitigate the risks associated with prebedtime screen use [63] and sleep interventions in general.

### Strengths, Limitations, and Future Research

A strength of this study was the measurement of adolescents’ screen exposure, which was significantly enhanced through the use of wearable cameras. Such devices offer an improvement over existing self-report measures of lifestyle behaviors and the contexts in which they occur. A high agreement between coders was reported, similar to a previous study using wearable cameras [39]. However, these devices also have limitations. First, the 10-second epoch between image capture may have missed possible screen exposure, particularly quick and sporadic smartphone checking [49]. Future studies that compare a continuous video or 1-second epoch with longer intervals between image capture are warranted. Second, if we wish to ascertain the function (eg, relaxation and entertainment) that different screens serve for adolescents, we are unlikely to do this through camera images. While we were able to infer the content being viewed, further qualitative work will enable a more in-depth understanding of what functions are being served by engaging in different devices and platforms. Third, there is the possibility of the Hawthorne effect, whereby participants modified their behavior in response to wearing an automated wearable camera. This may have implications for the validity of this study. Fourth, the annotation of wearable camera images was based on decisions made by the coders. This limitation may have been offset by conducting interrater reliability tests, showing an almost perfect agreement between coders [44]. The recent development of an annotation protocol for sedentary behavior in children using wearable cameras [64] shows promise, and once applied to larger samples of children, this protocol can help better understand adolescents’ contemporary screen engagement. In addition to coding issues, the data processing and coding times are limiting factors and may be unsuitable for use in large-scale studies, unless an automatic recognition algorithm is developed to classify different aspects of human behavior [26].

Other limitations include a small sample size and the relatively homogenous demographic characteristics of the sample. Therefore, the results are unlikely to be generalizable to the wider adolescent population. Future research needs to consider other sociodemographic groups to confirm the key findings observed in this study. A further limitation was that due to ethical concerns raised by school principals regarding camera wearing on school grounds, we only examined screen use during the after-school period and weekends. Since screen use before and during school may yield different results and patterns, future studies are needed to examine the exposure to screen use across the day. Finally, as with other wearable camera studies, the sample size was small, and thus, the study was insufficiently powered to use temporal patterns as a means for testing differences. This should be considered in future studies with larger sample sizes.

### Conclusion

Among a small sample of adolescents, we showed high amounts of screen use, most of which occurred in the home, with little social interaction. This information might be used when designing interventions to inform new policy to influence adolescents’ screen use. For example, Australian guidelines for physical activity and sedentary behavior recommend no more than two hours of recreational screen use daily for this age group [63]. Moreover, we showed that wearable cameras may provide a new approach to collect more accurate data on screen-based behaviors in free-living conditions, and with some volume. As such, we were able to both enhance traditional self-report and provide context and temporal specificity surrounding screen-based behaviors in free-living settings. Our findings may be used to inform guidelines and protocols for visual research on screen-based behaviors, and form a basis for larger-scale studies for comparisons.

## Appendix

Multimedia Appendix 1 Summary of the coding framework.

Multimedia Appendix 2 Frequency of images and camera wear time per day.

Multimedia Appendix 3 Description of multiscreening among adolescents.

Multimedia Appendix 4 Physical setting of adolescents’ screen-based activities.

Multimedia Appendix 5 Social setting and interaction of adolescents’ screen-based activities.

Multimedia Appendix 6 Co-existing behaviors of adolescents’ screen-based activities.
