# Supplementary material for: Using Wearable Cameras to Categorize the Type and Context of Screen-Based Behaviors Among Adolescents: Observational Study
Source: JMIR Pediatr Parent. 2022 Mar 21;5(1):e28208. doi: 10.2196/28208 (PMC8981006; doi:10.2196/28208)
Supplement: Multimedia Appendix 1 [file pediatrics_v5i1e28208_app1.docx]

**Multimedia Appendix 1.** Summary of the coding framework.

| **Device Attention** | **Device**  **Type** | **Content**  **Type^a^** | **Content Classification** | **Physical**  **Setting^b^** | **Social**  **Setting** | **Social**  **Interaction** | **Co-existing**  **Behaviours^c^** |
| --- | --- | --- | --- | --- | --- | --- | --- |
| Primary | Television | TV Programs | Recreational | Home | Alone | None | Eating |
| Secondary | Smartphone | Internet | Educational | Public | Adult | Co-participating | Multitasking |
| Background | Laptop computer | Gaming | Social | Transport | Child | Co-viewing |  |
|  | Tablet | Creative |  |  |  | Background |  |
|  | Desktop computer | Communication |  |  |  |  |  |
|  | Smartwatch | Social media |  |  |  |  |  |
|  |  | General |  |  |  |  |  |

^a^ **TV Programs:** refers to any form of TV show, movie, or video, including YouTube videos—*action*, *animation/cartoons*, *action animation*;

**Internet:** refers to all interest-based activities other than those for social media, gaming or watching online videos—*article/book/blog*, *browsing*;

**Gaming:** refers to playing a video game, including on gaming consoles via TV set—*action*, *simulation*, *strategy/puzzle*, *sports*;

**Creative:** refers to visual content on screen devices that has been created—*productivity software*, *art apps*, *camera apps*;

**Communication:** refers to screen media that is primarily used to communicate with other people—*instant/text messaging*, *call*, *video* *chat*, *email*;

**Social media:** refers to websites and apps that allow users to share information on the internet—*Instagram*, *Facebook*, *TikTok*, *Snapchat*;

**General**: refers to common features (e.g., home screen, notifications, lock screen, calculator).

^b^ **Home**: *living room*, *bedroom*, *kitchen/dining room*, *other* *(e.g., office)*;

**Public**: *community* *venue*, *retail*, *food* *retail*;

**Transport**: *public* *(e.g., bus, train)*, *private* *(e.g., car)*.

^c^ **Eating**: *meal*, *snack*, *beverage*;

**Multitask**: *hobby*, *writing*, *reading*
