# Supplementary material for: Using Wearable Cameras to Categorize the Type and Context of Screen-Based Behaviors Among Adolescents: Observational Study
Source: JMIR Pediatr Parent. 2022 Mar 21;5(1):e28208. doi: 10.2196/28208 (PMC8981006; doi:10.2196/28208)
Supplement: Multimedia Appendix 2 [file pediatrics_v5i1e28208_app2.docx]

**Multimedia Appendix 2.** Frequency of images and camera wear time per day.

| **Variable**^a^ | **Weekday** | | | | **Weekend** | | | |
| --- | --- | --- | --- | --- | --- | --- | --- | --- |
|  | **Mean** | **Minimum** | **Median** | **Maximum** | **Mean** | **Minimum** | **Median** | **Maximum** |
| Number of images | 1,365 | 291 | 1,314 | 2,674 | 3,025 | 1,306 | 2,905 | 4,500 |
| Time of first image, h:min:s | 16:11:42 | 14:24:42 | 15:57:47 | 19:47:37 | 08:19:12 | 05:52:59 | 08:06:12 | 13:29:03 |
| Time of last image, h:min:s | 20:40:15 | 17:27:16 | 20:47:56 | 00:24:07 | 20:40:15 | 15:11:36 | 21:11:14 | 11:30:50 |
| Wear time, min^b^ | 230.5 | 48.4 | 224.8 | 447.2 | 508.1 | 218.6 | 484.4 | 751.0 |
| Captured time, min^c^ | 227.5 | 48.5 | 219.0 | 445.7 | 504.2 | 217.7 | 484.2 | 750.0 |
| Screen images | 1,005 | 267 | 1,016 | 2,348 | 2,228 | 486 | 2,165 | 3,960 |
| Screen time, min^c^ | 167.5 | 44.5 | 169.3 | 391.3 | 371.3 | 81.0 | 360.8 | 660.0 |
| Absent or inactive screen images | 350 | 11 | 256 | 1463 | 802 | 228 | 436 | 2,160 |
| Blocked or blurry images | 1 | 0 | 0 | 21 | 0 | 0 | 0 | 4 |

^a^Included 40 days (30 weekday school evenings and 10 weekend days) from 10 participants

^b^Minutes the camera was turned on

^c^One image represents 10 seconds (number of images/6)

h, hour; min, minute; s, second
