# Supplementary material for: Using Wearable Cameras to Categorize the Type and Context of Screen-Based Behaviors Among Adolescents: Observational Study
Source: JMIR Pediatr Parent. 2022 Mar 21;5(1):e28208. doi: 10.2196/28208 (PMC8981006; doi:10.2196/28208)
Supplement: Multimedia Appendix 3 [file pediatrics_v5i1e28208_app3.docx]

**Electronic Supplementary Table 3. Description of Multiscreening among Adolescents**

| **Device Combination**^a^  *Content combination*^a^ | ***n* of images** | **%** |
| --- | --- | --- |
| **Total Images with Multiple Screens** | **11,976** | **16.8** |
| **Primary: TV Set, Background: Smartphone**  *Gaming and TV programs*  *TV programs and TV programs* | **5,029**  4,973  56 | **42.0**  41.5  0.5 |
| **Primary: Smartphone, Background: TV Set**  *TV programs and gaming*  *Social media and gaming*  *Social media and TV programs*  *Communicating and TV programs*  *Internet and TV programs*  *Creative and gaming*  *TV programs and TV programs*  *Gaming and gaming*  *Social media and unknown*  *Internet and gaming*  *Communicating and TV programs*  *Interactive and gaming* | **2,285**  725  465  336  180  157  119  85  83  62  49  16  8 | **19.1**  6.1  3.9  2.8  1.5  1.3  1.0  0.7  0.7  0.5  0.4  0.1  0.1 |
| **Primary: Smartphone, Background: Laptop**  *Social media and internet*  *Social media and creative*  *Communication and creative*  *Communication and internet*  *Unknown and creative*  *Unknown and internet*  *Social media and general*  *TV programs and general*  *Interactive and internet*  *General and internet*  *Creative and creative*  *Social media and TV programs*  *Communication and internet*  *Interactive and creative*  *General and general* | **1,465**  388  307  242  113  66  60  52  38  14  12  10  8  6  3  2 | **12.2**  3.2  2.6  2.0  0.9  0.6  0.5  0.4  0.3  0.1  0.1  0.1  0.1  0.1  0.0  0.0 |
| **Primary: Laptop, Background: TV Set**  *TV programs and TV programs*  *Creative and TV programs*  *Internet and TV programs* | **985**  780  194  11 | **8.2**  6.5  1.6  0.1 |
| **Primary: Laptop, Secondary: Smartphone**  *Creative and TV programs*  *Internet and TV programs*  *Creative and internet*  *Creative and social media*  *TV programs and TV programs* | **857**  578  130  101  34  14 | **7.2**  4.8  1.1  0.8  0.3  0.1 |
| **Primary: Laptop, Secondary: Tablet**  *Creative and TV programs*  *Internet and TV programs* | **525**  468  57 | **4.4**  3.9  0.5 |
| **Primary: Smartphone, Background: Smartphone**  *Gaming and general*  *Social media and general* | **268**  200  68 | **2.2**  1.7  0.6 |
| **Primary: Smartphone. Secondary: Laptop**  *Creative and social media*  *Communication and creative*  *Unknown and creative*  *Social media and creative*  *Gaming and TV programs*  *Interactive and creative*  *Communication and internet* | **236**  69  45  35  31  28  8  7 | **2.0**  0.6  0.4  0.3  0.3  0.2  0.1  0.1 |
| **Primary: Tablet, Secondary: Laptop**  *Internet and creative* | **124**  124 | **1.0**  1.0 |
| **Primary: Smartphone, Background: Tablet**  *Communication and creative*  *Communication and communication*  *Communication and internet*  *General and internet* | **69**  34  15  13  7 | **0.6**  0.3  0.1  0.1  0.1 |
| **Primary: Tablet, Background: Laptop**  *TV programs and creative*  *Internet and general*  *TV programs and general*  *Internet and internet*  *Creative and creative* | **58**  31  12  11  2  2 | **0.5**  0.3  0.1  0.1  0.0  0.0 |
| **Primary: Smartphone, Background: Desktop**  *Social media and unknown* | **16**  16 | **0.1**  0.1 |
| **Primary: Smartphone, Secondary: TV Set**  *Internet and gaming* | **10**  10 | **0.1**  0.1 |
| **Primary: Smartphone, Secondary: Smartphone**  *General and general* | **8**  8 | **0.1**  0.1 |
| **Primary: Unclassified, Background: Laptop**  *Unknown and creative* | **5**  5 | **0.0**  0.0 |
| **Primary: Smartphone, Background: Laptop & TV Set**  *Communication, internet, and TV programs*  *Social media, creative, and TV programs*  *Social media, internet and TV programs* | **23**  12  6  5 | **0.2**  0.1  0.1  0.0 |
| **Primary: Smartphone, Background: Tablet & Laptop**  *Social media, TV programs and creative* | **13**  13 | **0.1**  0.1 |

^a^ Frequency and proportion of screens in total image set (71,936 images)
