# Supplementary material for: Using Wearable Cameras to Categorize the Type and Context of Screen-Based Behaviors Among Adolescents: Observational Study
Source: JMIR Pediatr Parent. 2022 Mar 21;5(1):e28208. doi: 10.2196/28208 (PMC8981006; doi:10.2196/28208)
Supplement: Multimedia Appendix 4 [file pediatrics_v5i1e28208_app4.docx]

**Multimedia Appendix 4.** Physical setting of adolescents’ screen-based activities.

| **Device**  *Location*^a^ | ***n* of images** | **%** |
| --- | --- | --- |
| **All Screens**^b^  *Living Room*  *Bedroom*  *Kitchen/Dining Room*  *Private Transport*  *Other (e.g., Home office)*  *Public Transport*  *Public Food Outlet*  *Community Venue* | **64,856**  37,364  19,473  4,703  1,564  915  330  326  181 | 57.6  30.0  7.3  2.4  1.4  0.5  0.5  0.3 |
| **TV Set**  *Living Room*  *Bedroom*  *Kitchen/Dining Room*  **TV Set: Action Gaming**  *Living Room*  **TV Set: TV-Viewing**  *Living Room*  *Bedroom*  *Kitchen/Dining Room*  **Unclassifiable**  *Living room* | **25,950**  24,940  852  158  **14,032**  14,032  **11,803**  10,793  852  158  **115**  115 | 96.1  3.3  0.6  100.0  91.4  7.2  1.4  100.0 |
| **Smartphone**  *Living Room*  *Bedroom*  *Kitchen/Dining Room*  *Private Transport*  *Public Food Outlet*  *Public Transport*  *Other (Office, Bathroom)*  *Public Community Venue* | **20,851**  8,719  7,932  1,697  1,564  326  241  191  181 | 41.8  38.0  8.1  7.5  1.6  1.2  0.9  0.9 |
| **Laptop Computer**  *Bedroom*  *Living Room*  *Kitchen/Dining Room*  *Other (Office, Bathroom)*  *Public Transport* | **15,309**  8,974  3,705  2,000  541  89 | 58.6  24.2  13.1  3.5  0.6 |
| **Tablet**  *Bedroom*  *Kitchen/Dining Room*  *Other (Office, Bathroom)* | **2,720**  1,709  848  163 | 62.8  31.2  6.0 |
| **Desktop Computer**  *Other (Office, Bathroom)* | **20**  20 | 100.0 |
| **Wearable Smartwatch**  *Bedroom* | **1**  1 | 100.0 |
| **Unclassifiable**  *Bedroom* | **5**  5 | 100.0 |

^a^ Frequency and proportion of images nested within individual screen domains (e.g., TV)

^b^ Based on all screen-based coding interactions (including images with multiple screens)
