# Supplementary material for: Using Wearable Cameras to Categorize the Type and Context of Screen-Based Behaviors Among Adolescents: Observational Study
Source: JMIR Pediatr Parent. 2022 Mar 21;5(1):e28208. doi: 10.2196/28208 (PMC8981006; doi:10.2196/28208)
Supplement: Multimedia Appendix 5 [file pediatrics_v5i1e28208_app5.docx]

**Multimedia Appendix 5.** Social setting and interaction of adolescents’ screen-based activities.

| **Device**  **Social Interaction**^a^  *Type of Social Interaction*^a^ | ***n* of images** | **%** |
| --- | --- | --- |
| **All Screens**^b^  **Alone**  **Adult**  *Background*  *Co-viewing*  *Co-participating*  **Child**  *Background*  *Co-viewing* | **64,856**  **54,430**  **6,867**  4,943  1,917  7  **3,559**  2,155  1,404 | **83.9**  **10.6**  7.6  3.0  0.0  **5.5**  3.3  2.2 |
| **TV Set**  **Alone**  **Adult**  *Co-viewing*  *Background*  **Child**  *Background*  *Co-viewing*  **TV Set: Action Gaming**  **Alone**  **Adult**  *Background*  **Child**  *Background*  **TV Set: TV-Viewing**  **Alone**  **Adult**  *Co-viewing*  *Background*  **Child**  *Background*  *Co-viewing*  **Unclassifiable**  **Adult**  *Co-viewing*  *Background* | **25,950**  **20,334**  **2,983**  1,778  1,205  **2,633**  1,265  1,368  **14,032**  **12,915**  **501**  501  **616**  616  **11,803**  **7,419**  **2,367**  1,719  648  **2,017**  649  1,368  **115**  **115**  59  56 | **78.4**  **11.5**  6.9  4.6  **10.1**  4.9  5.3  **92.0**  **3.6**  3.6  **4.4**  4.4  **62.9**  **20.0**  14.5  5.5  **17.1**  5.5  11.6  **100.0**  51.3  48.7 |
| **Smartphone**  **Alone**  **Adult**  *Background*  *Co-viewing*  **Child**  *Background*  *Co-viewing* | **20,851**  **17,372**  **2,753**  2,668  85  **726**  720  6 | **83.3**  **13.2**  12.8  0.4  **3.5**  3.5  0.0 |
| **Laptop Computer**  **Alone**  **Adult**  *Background*  *Co-participating*  **Child**  *Background*  *Co-viewing* | **15,309**  **14,087**  **1,077**  1,070  7  **145**  115  30 | **92.0**  **7.0**  7.0  0.0  **1.0**  0.8  0.2 |
| **Tablet**  **Alone**  **Adult**  *Co-viewing*  **Child**  *Background* | **2,730**  **2,631**  **34**  34  **55**  55 | **96.7**  **1.3**  1.3  **2.0**  2.0 |
| **Desktop Computer**  **Adult**  *Co-viewing* | **20**  **20**  20 | **100.0**  100.0 |
| **Wearable Smartwatch**  **Alone** | **1**  **1** | **100.0** |
| **Unclassifiable**  **Alone** | **5**  **5** | **100.0** |

^a^ Frequency and proportion of images nested within individual screen domain (e.g., TV)

^b^ Based on all screen-based coding interactions (including images with multiple screens)
