# Supplementary material for: Using Wearable Cameras to Categorize the Type and Context of Screen-Based Behaviors Among Adolescents: Observational Study
Source: JMIR Pediatr Parent. 2022 Mar 21;5(1):e28208. doi: 10.2196/28208 (PMC8981006; doi:10.2196/28208)
Supplement: Multimedia Appendix 6 [file pediatrics_v5i1e28208_app6.docx]

**Multimedia Appendix 6.** Co-existing behaviors of adolescents’ screen-based activities.

| **Device**  *Co-existing Behaviours*^a^ | ***n* of images** | **%** |
| --- | --- | --- |
| **All Screens**^b^  *None*  *Pen and Paper*  *Snack*  *Meal*  *Hobby*  *Beverage*  *Other*  *Book* | **64,856**  56,656  3,873  1,755  1,454  602  297  185  34 | 87.4  6.0  2.7  2.2  0.9  0.5  0.3  0.1 |
| **TV Set**  *None*  *Meal*  *Snack*  *Hobby*  *Beverage*  *Pen and Paper*  *Book*  *Other*  **TV Set: Action Gaming**  *None*  *Snack*  *Pen and Paper*  *Beverage*  **TV Set: TV-Viewing**  *None*  *Meal*  *Hobby*  *Snack*  *Beverage*  *Book*  *Other*  **Unclassifiable**  *None* | **25,950**  23,461  966  791  487  198  29  10  8  **14,032**  13,514  469  29  20  **11,803**  9,832  966  487  322  178  10  8  **115**  115 | 90.4  3.7  3.1  1.9  0.8  0.1  0.0  0.0  96.3  3.3  0.2  0.2  83.3  8.2  4.1  2.7  1.5  0.1  0.1  100.0 |
| **Smartphone**  *None*  *Pen and Paper*  *Snack*  *Meal*  *Hobby*  *Beverage*  *Other*  *Book* | **20,851**  19,003  614  569  429  115  99  14  8 | 91.1  2.9  2.7  2.1  0.6  0.5  0.1  0.0 |
| **Laptop Computer**  *None*  *Pen and Paper*  *Snack*  *Meal*  *Book* | **15,309**  12,673  2,430  131  59  16 | 82.8  15.9  0.8  0.4  0.1 |
| **Tablet**  *None*  *Pen and Paper*  *Snack*  *Other* | **2,720**  1,493  800  264  163 | 54.9  29.4  9.7  6.0 |
| **Desktop Computer**  *None* | **20**  20 | 100.0 |
| **Wearable Smartwatch**  *None* | **1**  1 | 100.0 |
| **Unclassifiable**  *None* | **5**  5 | 100.0 |

^a^ Frequency and proportion of images nested within individual screen domains (e.g., TV)

^b^ Based on all screen-based coding interactions (including images with multiple screens)
